# Assessing the Quality of Life of Parents of Children With Thalassemia: A Cross-Sectional Study in Medina City, Saudi Arabia

**DOI:** 10.7759/cureus.71653

**Published:** 2024-10-16

**Authors:** Muhammad A Tobaiqi, Abdullah H Aljohani, Feras S Alharbi, Uthman M Alshenqiti, Nawaf M Alahmadi, Mohamed A Abdelrahman, Mazen K Almughamsi, Nadeem A Ansari, Muayad S Albadrani

**Affiliations:** 1 College of Medicine, Taibah University, Medina, SAU; 2 Department of Family and Community Medicine, College of Medicine, Taibah University, Medina, SAU

**Keywords:** children, kingdom of saudi arabia (ksa), medina, parents, quality of life, thalassemia

## Abstract

Background: Thalassemia is a group of genetic disorders that result in a lack of hemoglobin (Hb) production. Children with thalassemia rely on regular blood transfusions for survival, which places a significant burden on their families and negatively impacts their quality of life (QOL).

Objective: This study aimed to assess QOL from the perspective of parents of thalassemic children in Medina City, Saudi Arabia.

Method: A cross-sectional study was conducted among 52 parents in Medina through September and October 2023 using a socio-demographic and clinical characteristic questionnaire and an assessment of parents’ QOL.

Results: The study was conducted among 52 parents in Medina, with a mean (standard deviation (SD)) age of 44.4 (9.5). Most participants (63.5%) were males, 90.4% were married, and 61.5% had one child with thalassemia. The majority (84.6%) had a blood transfusion every three weeks, and 80.8% had Hb before transfusion less than 9 gm/dL. The mean (SD) QOL and health score was 246.4 (51.2) out of 400. The parent’s low income (p<0.001) and being non-Saudi parents (p=0.017) were significant factors for low QOL and health.

Conclusion: Parents with thalassemic children revealed moderate QOL and health in Medina City, Saudi Arabia. Low income and expatriation negatively affected the parents’ QOL and health. These results emphasize the need for financial support through assistance programs to alleviate the financial burden on these families. Additionally, specialized thalassemia treatment centers should be established to ensure easy access to comprehensive healthcare services.

## Introduction

Thalassemia is a group of chronic genetic blood disorders. It is prevalent in several regions, including the Middle East, Southeast Asia, the Mediterranean, sub-Saharan Africa, and the Indian subcontinent. According to a prior study, the percentage of thalassemia carriers and thalassemia-affected individuals in Saudi Arabia was 3.22% and 0.7%, respectively [1]. Moreover, beta thalassemia is more prevalent in the eastern region of Saudi Arabia (5.9%) than in the other regions [2]. In developing nations, the high number of children born with thalassemia is often caused by a combination of several factors. These factors include consanguineous marriages, high birth and fertility rates, early marriages, and low levels of education. Moreover, a lack of awareness about the thalassemia problem exacerbates the situation [3].

Thalassemia is attributed to gene mutations responsible for producing hemoglobin (Hb), a protein found in red blood cells (RBCs). These mutations result in abnormal globin gene expression, which leads to the absence of globin chain synthesis. There are two main subtypes of thalassemia, alpha-thalassemia and beta-thalassemia, named after the subunit of the affected globin chain. Alpha-thalassemia is typically caused by deletions within the alpha-globin gene. In contrast to alpha-thalassemia, beta-thalassemia is usually due to point mutations involving only one or two beta nucleotides. As a result, beta-thalassemia patients can be classified as either beta-thalassemia major, intermedia, or minor based on their genetic background and clinical presentation [4,5].

Abnormal RBCs with lower Hb concentrations are produced when a globin chain is missing, leading to precipitation of excess globin chains and shortened survival of RBCs through hemolysis. Additionally, ineffective erythropoiesis will occur, which is the premature death of RBC precursors that takes place within the bone marrow, leading to chronic anemia. This condition is often accompanied by massive erythroid hyperplasia, which causes the hematopoiesis process to spread to other body parts, including the skull, resulting in facial bone expansion (also known as chipmunk facies). Over time, extramedullary hematopoiesis occurs, leading to hepatosplenomegaly [6].

Thalassemia can affect physical appearance due to bone marrow expansion, leading to skeletal deformities, osteoporosis, and short stature, negatively impacting self-image. It may also cause health issues such as growth problems, retardation, and delayed puberty. Patients with severe thalassemia often experience other serious complications, including heart failure, cardiac arrhythmia, liver disease, hepatosplenomegaly, endocrine complications (hypogonadism, hypothyroidism, diabetes, hypoparathyroidism), and infections [7]. The patient's social behavior and psychology may be affected by this disease, depending on the type of Hb level [8,9]. Due to the vast range in clinical severity, some individuals may not require any treatment, while others may need regular blood transfusions and iron chelation therapy to remove the excess iron from their bodies. The accumulation of excess iron in the body is the primary factor that can cause health issues and even lead to death in people with thalassemia [10].

In addition, the quality of life (QOL) concept is increasingly important for those with chronic illnesses, including patients and caregivers. According to the World Health Organization (WHO), QOL is the individual's perception of their position in life concerning their goals, expectations, standards, concerns, and the cultural and value systems in which they reside [11]. Thus, QOL is especially important for chronic and debilitating conditions like thalassemia [12].

The chronic nature of thalassemia in children can cause various psychosocial issues for their families. The stress experienced by the families of thalassemia patients might negatively affect their QOL. Parents of thalassemic children may face disappointment, uncertainty, anxiety, depression, worries about school performance, troubles with the job and medical care, welfare concerns, culture, family, and economic difficulties. [13,14]. Studies have been conducted to examine the QOL and health of individuals with thalassemia. However, most of these studies focus on the affected individuals themselves rather than their parents [15-17]. There is little information available on the QOL for parents of children with thalassemia, particularly in certain regions [12,18]. A child with a chronic disease like thalassemia, which requires life-long treatment, can have a significant psychological impact on the family. Therefore, it is important to investigate the different ways this could affect the family and develop improvement strategies. 

## Materials and methods

Study design

This cross-sectional study was performed among parents in Medina, Saudi Arabia. The connection with the Al-Medina Hereditary Blood Disorder Charity Society was used to recruit participants. The data collection was from September to October 2023.

Study population

The study included parents in Medina City, Saudi Arabia, whose children had thalassemia and had not been cured of it. Parents from other regions than Al-Medina and children who had bone marrow transplants and were cured of thalassemia were excluded from the study.

Sampling technique

We followed a registry-based sampling technique as this is a rare disease. Parents of all children with thalassemia registered in the Al-Medina Hereditary Blood Disorder Charity Society and, following the study eligibility criteria of having uncured thalassemia, agreed to participate.

Data collection

Data was collected through a structured, validated questionnaire [11]. The questionnaire was self-administrated and validated through translation from English to Arabic. The data collection tool consisted of three sections. The first section included the participants' demographic characteristics such as age, gender, nationality, educational level, social status, family member number, history of death due to thalassemia in the family, and thalassemic child order in the family. The second section included the participants’ clinical characteristics, such as frequency of blood transfusion, Hb level before blood transfusion, serum ferritin levels, history of surgical splenectomy, and iron chelator usage. The third section included the WHOQOL-BREF (The World Health Organization Quality of Life Brief Version) questionnaire. Regarding the WHOQOL-BREF questionnaire, there are 26 items divided into four domains related to QOL: physical health, psychological health, social relationships, and environment, and individual items covering overall QOL and general health. Higher scores indicate better QOL and health.

Data entry and analysis

The data was extracted and revised in an Excel sheet. Statistical analysis was conducted using IBM SPSS Statistics for Windows, Version 26 (Released 2019; IBM Corp., Armonk, New York, United States). Categorical variables were described in numbers and percentages. Continuous, non-normally distributed variables were reported as the median and interquartile range (IQR). The score was calculated as a scale from 1 to 5 for each domain, then transformed to a 0-100 scale as per the validated questionnaire [11]. The total score was the sum of four domains. The association between WHOQOL-BREF scores and independent variables was conducted using the Mann-Whitney test. P-values less than 0.05 were considered statistically significant.

Ethical considerations

This research was approved by the College of Medicine Institutional Review Board at Taibah University with ethical ID number "STU-22-025". All methods were performed in accordance with the relevant guidelines and regulations (e.g. Declaration of Helsinki). Informed consent to participate was obtained from each participant.

## Results

The study included 52 participants aged 29 to 65 years with a mean (standard deviation [SD]) age of 44.4 (9.5). Most participants (63.5%) were males, 90.4% were married, and 61.5% had one child with thalassemia. 36.5% of the participants were Saudi, and 32.7% had a university degree. More than half of the participants (57.7%) were employees; 53.8% had more than five members in their family and had low income. About one-third of the parents had more than one child affected by thalassemia. Additionally, a small proportion of the participants (17.3%) had a death case of thalassemia in the family, as shown in Table 1.

**Table 1:** Demographic characteristics of the participants (N=52) SD: Standard deviation; IQR: Interquartile range; N: Number

Age (Years)	Mean (SD)	44.4 (9.5)	
	Median (IQR)	42 (14)	
	Min-Max	29-65	
Parameters	Category	N	Percentage
Gender	Male	33	63.5
	Female	19	36.5
Nationality	Saudi	19	36.5
	Egyptian	5	9.6
	Palestinian	3	5.8
	Afghanistan	1	1.9
	Chinese	1	1.9
	Yemeni	4	7.7
	Pakistani	9	17.3
	Syrian	3	5.8
	Myanmar	6	11.5
Educational level	Primary	11	21.2
	Intermediate	9	17.3
	High school	15	28.8
	University	17	32.7
Marital status	Single	5	9.6
	Married	47	90.4
Monthly income	Low	28	53.8
	Middle	24	46.2
Parents employment status	Employee	30	57.7
	Unemployed	22	42.3
Family members	Equal to or less than five members	24	46.2
	More than five members	28	53.8
Number of affected children in the family	1	32	61.5
	2	13	25.0
	3	5	9.6
	5	2	3.8
Is there a death due to thalassemia in the family	Yes	9	17.3
	No	43	82.7
Thalassemic child sequence in the family	1^st^	16	30.8
	2^nd^	16	30.8
	3^rd^	5	9.6
	4^th^	4	7.7
	5^th^	4	7.7
	6^th^	1	1.9
	7^th^	2	3.8
	8^th^	2	3.8
	10^th^	1	1.9
	13^th^	1	1.9

Regarding the clinical characteristics of the participants’ children, their serum ferritin had a mean (SD) level of 4090.7 (2511.4), ranging from 969 to 9000 μg/L. Additionally, 84.6% had a blood transfusion every three weeks, and 80.8% had an Hb level of less than 9 gm/dL before the transfusion. Moreover, 40.4% of children had a history of splenectomy, and 7.6% had complications of high Hb. However, 63.5% had no complications. Half of the participants received deferoxamine as an iron chelator, as shown in Table 2.

**Table 2:** Clinical characteristics of the participants (N=52) SD: Standard deviation; IQR: Interquartile range; N: Number

Serum ferritin level (μg/L) N=35	Mean (SD)	4090.7 (2511.4)	
	Median (IQR)	4000 (4000)	
	Min-Max	969-9000	
Parameters	Category	N	Percentage
Treating hospital	Royal Commission Medical Center	1	1.9
	Medical Care Center	1	1.9
	King Abdullah Medical City Specialist Hospital	1	1.9
	Al Bahah King Fahad Hospital	1	1.9
	Madinah King Fahd Hospital	28	53.8
	Kings Faisal Specialty Hospital	2	3.8
	Madinah Maternity and Children Hospital	18	34.6
Blood transfusion frequency	Every three weeks	44	84.6
	Every four weeks	4	7.7
	Every five weeks	4	7.7
Hb before transfusion	Less than 9 gm/dL	42	80.8
	More than 9 gm/dL	10	19.2
History of splenectomy	Yes	21	40.4
	No	31	59.6
Complications	High Hb	4	7.6
	Diabetes	2	3.8
	Liver disorder	2	1.9
	Constant fatigue, exhaustion, and bad mood	2	3.8
	Friction of the thigh separation and the process of bitterness	1	1.9
	Shortness of breath and sudden headache	1	1.9
	Gallstones	1	1.9
	Rheumatic fever and use of pressure therapy	1	1.9
	In the reproductive system	1	1.9
	Yes, without specify	4	7.7
	No complications	33	63.5
History of using deferoxamine as an iron chelator	Yes	26	50
	No	26	50
Is there another infected child in the family	Yes	15	28.8
	No	37	71.2

Table 3 illustrates the participants’ responses to the WHOQOL-BREF questionnaire. One-third of the participants, 34.6%, responded that they don’t get support from others at all. Half of the participants reported that they had good QOL (51.9%). Additionally, 65.4% were satisfied with their health.

**Table 3:** Percentage of participants’ responses to the WHOQOL-BREF questionnaire WHOQOL-BREF: The World Health Organization Quality of Life Brief Version

Question	Response	N	Percentage
Do you get the kind of support that you need from others?	-Not at all	18	34.6
	-Slightly	12	23.1
	-Moderately	16	30.8
	-Completely	5	9.6
	-Very	1	1.9
How would you rate your QOL?	-Very poor	2	3.8
	-Poor	16	30.8
	-Neither poor nor good	2	3.8
	-Good	27	51.9
	-Very good	5	9.6
How satisfied are you with your health?	-Fairly dissatisfied	2	3.8
	-Neither satisfied nor dissatisfied	6	11.5
	-Satisfied	34	65.4
	-Very satisfied	10	19.2
To what extent do you feel that physical pain prevents you from doing what you need to do?	-Not at all	10	19.2
	-A small amount	17	32.7
	-A moderate amount	17	32.7
	-A great deal	8	15.4
How much do you need any medical treatment to function in your daily life?	-Not at all	9	17.3
	-A small amount	14	26.9
	-A moderate amount	17	32.7
	-A great deal	11	21.2
	-An extreme amount	1	1.9
How much do you enjoy life?	-Not at all	3	5.8
	-A small amount	9	17.3
	-A moderate amount	24	46.2
	-A great deal	13	25.0
	-An extreme amount	3	5.8
To what extent do you feel your life to be meaningful?	-Not at all	2	3.8
	-A small amount	11	21.2
	-A moderate amount	15	28.8
	-A great deal	20	38.5
	-An extreme amount	4	7.7
How well are you able to concentrate?	-Not at all	1	1.9
	-Slightly	7	13.5
	-Moderately	22	42.3
	-Very	20	38.5
	-Extremely	2	3.8
How safe do you feel in your daily life?	-Not at all	1	1.9
	-Slightly	7	13.5
	-Moderately	16	30.8
	-Very	25	48.1
	-Extremely	3	5.8
How healthy is your physical environment?	-Not at all	1	1.9
	-Slightly	9	17.3
	-Moderately	25	48.1
	-Very	16	30.8
	-Extremely	1	1.9
Do you have enough energy for everyday life?	-Not at all	2	3.8
	-Slightly	11	21.2
	-Moderately	21	40.4
	-Very	16	30.8
	-Extremely	2	3.8
Are you able to accept your bodily appearance?	-Not at all	1	1.9
	-Slightly	3	5.8
	-Moderately	14	26.9
	-Very	27	51.9
	-Extremely	7	13.5
Do you have enough money to meet your needs?	-Not at all	13	25.0
	-Slightly	15	28.8
	-Moderately	21	40.4
	-Very	3	5.8
	-Extremely	13	25.0
How much information you need in your daily life is available to you?	-Not at all	5	9.6
	-Slightly	9	17.3
	-Moderately	23	44.2
	-Very	12	23.1
	-Extremely	3	5.8
To what extent do you have the opportunity for leisure activities?	-Not at all	8	15.4
	-Slightly	15	28.8
	-Moderately	19	36.5
	-Very	8	15.4
	-Extremely	2	3.8
How well are you able to get around physically?	-Not at all	1	1.9
	-Slightly	18	34.6
	-Moderately	6	11.5
	-Very	23	44.2
	-Extremely	4	7.7
How satisfied are you with your sleep?	-Very dissatisfied	3	5.8
	-Fairly dissatisfied	10	19.2
	-Neither satisfied nor dissatisfied	29	55.8
	-Satisfied	10	19.2
	-Very satisfied	3	5.8
How satisfied are you with your ability to perform your daily living activities?	-Very dissatisfied	1	1.9
	-Fairly dissatisfied	5	9.6
	-Neither satisfied nor dissatisfied	8	15.4
	-Satisfied	30	57.7
	-Very satisfied	8	15.4
How satisfied are you with your capacity for work?	-Very dissatisfied	5	9.6
	-Fairly dissatisfied	10	19.2
	-Neither satisfied nor dissatisfied	32	61.5
	-Satisfied	5	9.6
	-Very satisfied	5	9.6
How satisfied are you with yourself?	-Very dissatisfied	3	5.8
	-Fairly dissatisfied	4	7.7
	-Neither satisfied nor dissatisfied	28	53.8
	-Satisfied	17	32.7
	-Very satisfied	3	5.8
How satisfied are you with your personal relationships?	-Fairly dissatisfied	3	5.8
	-Neither satisfied nor dissatisfied	5	9.6
	-Satisfied	32	61.5
	-Very satisfied	12	23.1
How satisfied are you with your sex life?	-Very dissatisfied	2	3.8
	-Fairly dissatisfied	2	3.8
	-Neither satisfied nor dissatisfied	18	34.6
	-Satisfied	22	42.3
	-Very satisfied	8	15.4
How satisfied are you with the support you get from your friends?	-Very dissatisfied	3	5.8
	-Fairly dissatisfied	4	7.7
	-Neither satisfied nor dissatisfied	19	36.5
	-Satisfied	23	44.2
	-Very satisfied	3	5.8
How satisfied are you with the conditions of your living place?	-Very dissatisfied	3	5.8
	-Fairly dissatisfied	5	9.6
	-Neither satisfied nor dissatisfied	10	19.2
	-Satisfied	25	48.1
	-Very satisfied	9	17.3
How satisfied are you with your access to health services?	-Very dissatisfied	2	3.8
	-Fairly dissatisfied	5	9.6
	-Neither satisfied nor dissatisfied	10	19.2
	-Satisfied	31	59.6
	-Very satisfied	4	7.7
How satisfied are you with your transport?	-Very dissatisfied	3	5.8
	-Fairly dissatisfied	4	7.7
	-Neither satisfied nor dissatisfied	5	9.6
	-Satisfied	34	65.4
	-Very satisfied	6	11.5
How often do you have negative feelings such as blue mood, despair, anxiety, or depression?	-Always	5	9.6
	-Frequently	7	13.5
	-Sometimes	19	36.5
	-Infrequently	17	32.7
	-Never	4	7.7

Regarding physical health domain questions, 32.7% felt moderate pain, the other 32.7% felt a small amount of physical pain, and 32.7% needed moderate medical treatment to function in their daily life. Additionally, 40.4% had moderately enough energy for everyday life, and 44.2% were very able to get around physically. More than half of the participants were neither satisfied nor dissatisfied with their sleep (55.8%) and their capacity for work (61.5%).

Regarding psychological domain questions, 46.2% enjoyed their life by a moderate amount. Additionally, 38.5% felt a great deal that their life was meaningful. More than half of the participants were satisfied with their ability to perform their daily living activities (57.7%). Around 36.5% sometimes had negative feelings such as blue mood, despair, anxiety, or depression.

Regarding social relationships domain questions, 42.3% were satisfied with their sex life, 44.2% were satisfied with the support they received from their friends, and more than half of the participants (61.5%) were satisfied with their personal relationships.

Regarding environmental domain questions, about half of the participants (48.1%) felt very safe in their daily lives, moderately healthy in their physical environment, and satisfied with the conditions of their living place. Additionally, 40.4% had moderately enough money to meet their needs, 44.2% moderately had the availability of information they need in their daily life, and 36.5% had moderate opportunities for leisure activities. Moreover, 59.6% were satisfied with their access to health services, and 65.4% were satisfied with their transport.

Table 4 describes the total scores of the WHOQOL-BREF questions regarding thalassemia. The mean (SD) scores of the physical health, psychological, social relationship, and environmental domains were 62.9 (15.2), 61.1 (16.5), 66.7 (15.2), and 55.6 (15.7) out of 100, respectively. The mean (SD) score of total QOL and health was 246.4 (51.2) out of 400.

**Table 4:** The scores of the WHOQOL-BREF questions WHOQOL-BREF: The World Health Organization Quality of Life Brief Version

Parameter	Category	Mean (SD)	Median (IQR)
WHOQOL-BREF	Domain 1 (Physical health)	62.9 (15.2)	63 (13)
	Domain 2 (Psychological)	61.1 (16.5)	63 (25)
	Domain 3 (Social relationships)	66.7 (15.2)	69 (19)
	Domain 4 (Environment)	55.6 (15.7)	56 (25)
Quality of Life and Health	Total score	246.4 (51.2)	250 (82)

Factors influencing the physical health (domain 1), psychological health (domain 2), social relationships (domain 3), and environment (domain 4) were evaluated. For physical health, parents with a middle income had significantly higher scores than those with a low income (p=0.005), with mean (SD) scores of 69.4 (14.6) and 57.4 (13.6), respectively.

In the psychological domain, parents holding a university degree had significantly higher (p=0.023) psychological scores compared to those with less than a university degree, with mean (SD) scores of 68.5 (17.2) vs 57.5 (15.2). Similarly, parents with a middle income scored higher (p<0.001) than those with a low income, with mean (SD) scores of 71.1 (12.6) vs 52.5 (14.6).

Significant correlations were observed for social relationships with nationality (p=0.023) and monthly income (p=0.002). Saudi parents had higher social relationship scores than non-Saudi parents, with mean (SD) scores of 73.4 (12.5) vs 62.9 (15.4). Parents with a middle income also scored higher in this domain than those with a low income, with mean (SD) scores of 71.8 (16.5) vs 62.3 (12.6). No significant correlations were found between other demographic and clinical characteristics and social relationship scores.

In the environmental domain, significant correlations were identified with monthly income (p<0.001), the occurrence of death in the family due to thalassemia (p=0.002), and the hospital where the children were treated (p=0.009). Parents with a middle income had higher scores than those with low income, with mean (SD) scores of 63.7 (14.5) vs 48.7 (13.3). Parents with no family deaths due to thalassemia had higher scores than those with such deaths, with mean (SD) scores of 57.6 (16.2) vs 46.0 (7.3). Additionally, parents whose children were treated at a hospital in Madinah had lower environmental scores compared to others, with mean (SD) scores of 53.6 (15.3) vs 71.0 (8.2). No significant correlations were found between other demographic and clinical characteristics and environmental scores.

Regarding the QOL and health, there was a significant correlation between the QOL and health score with nationality (p=0.017) and parent’s monthly income (p<0.001). The Saudi parents had higher QOL and health scores than non-Saudi parents, with a mean (SD) of 268.5 (50.1) vs 233.6 (48.0), respectively. Additionally, parents with middle income had higher QOL and health scores than those with low income, with a mean (SD) of 276.1 (48.2) vs 220.9 (38.9), respectively. Other demographic and clinical characteristics had no significant correlation with the QOL and health score, as shown in Table 5.

**Table 5:** Correlation between the individuals’ characteristics and quality of life and health score of WHOQOL-BREF The Mann-Whitney test was used. WHOQOL-BREF: The World Health Organization Quality of Life Brief Version

Factors		Mean (SD)	P-value
Demographic			
Age	≤45 years	246.8 (56.0)	0.947
	>45 years	245.8 (45.6)	
Gender	Male	248.7 (51.7)	0.665
	Female	242.3 (51.4)	
Nationality	Saudi	268.5 (50.1)	0.017
	Non-Saudi	233.6 (48.0)	
Educational level	Less than a university degree	240.3 (45.4)	0.223
	University degree	258.9 (61.0)	
Marital status	Single	259.2 (59.6)	0.561
	Married	245.0 (50.7)	
Income	Low	220.9 (38.9)	<0.001
	Middle	276.1 (48.2)	
Parents work	Work	248.8 (56.4)	0.693
	Does not work	243.0 (44.1)	
Family members	Equal to or less than five members	242.6 (50.5)	0.576
	More than five members	250.7 (52.7)	
Number of affected children in the family	Only one child	252.9 (51.6)	0.248
	More than one child	235.9 (50.0)	
Is there a death due to thalassemia in the family	Yes	234.6 (35.5)	0.452
	No	248.8 (53.9)	
Thalassemic child sequence in the family	1^st ^or 2^nd^	236.4 (53.7)	0.075
	3^rd ^or more	262.3 (43.4)	
Treating hospital	King Fahd Hospital/ Maternity and Children Hospital (in Madinah)	242.0 (52.5)	0.092
	Others	279.5 (21.7)	
Serum ferritin	≤2500	229.6 (48.7)	0.134
	>2500	253.2 (51.2)	
Blood transfusion frequency	Every three weeks	245.3 (52.8)	0.727
	Every four or five weeks	252.2 (43.6)	
Hb before transfusion	Less than 9 gm/dL	249.3 (53.0)	0.408
	More than 9 gm/dL	234.2 (43.1)	
History of splenectomy	Yes	241.9 ((47.5)	0.606
	No	249.4 (54.1)	
Complications	No complications	241.1 (47.6)	0.332
	Complications	255.5 (57.1)	
History of using deferoxamine as an iron chelator	Yes	248.1 (52.1)	0.812
	No	244.6 (51.2)	
Is there another infected child in the family	Yes	234.3 (51.4)	0.282
	No	251.3 (51.0)	

## Discussion

This is the first study in the Al Medina region to evaluate the QOL and health of parents with thalassemic children. This study showed that parents with thalassemic children reported moderate QOL and health. A previous study conducted among parents of thalassemic showed that they exhibited poor QOL and health [12]. The reason could be attributed to inadequate parental knowledge about thalassemia [18]. Another study conducted among thalassemic children proved a significant negative impact of thalassemia and its treatment in terms of physical functioning and psychosocial functioning [16]. A previous study among thalassemic children that used supportive therapies, such as blood transfusions and iron chelation, showed improved psychosocial health and social functioning [17].

Additionally, our results demonstrate a correlation between the different QOL domains and various demographic and personal information. These findings suggest that low socioeconomic status and expatriation were associated with low QOL and health scores. In terms of economic status, the parents with middle income scored higher than those with low income, a finding also reported in the previous study in India [12]. Thus, there is a clear correlation between low economic status and poorer QOL and health, supported by existing literature. This correlation may relate to the inability to afford treatment or lower living standards, but it must be clarified. Another significant correlation is between nationality QOL and health. Saudi parents scored higher than non-Saudi parents; this could also be ascribed to the fact that not all of the non-Saudi population is eligible for treatment. It is worth noting that, aside from the location of the treating center, there was no correlation between the clinical characteristics of the children and QOL and health, whereas there was a significant correlation between different personal information with low scores in different QOL domains. One study has suggested no correlation between desferrioxamine and QOL [17], and other studies have shown no correlation between serum ferritin and QOL [15,16]. This data showed that the disease process of thalassemia in children by itself has limited if any, effects on the QOL of their parents. Although a previous study showed better social life satisfaction in adult patients with thalassemia, this study revealed low scores in social life for the parents [15]. This may indicate that parents’ social life is affected more by the illness in their children than by their illness. Therefore, improving the quality of patient care positively impacts their overall QOL. In addition to medical care, patients may benefit from receiving socioeconomic support to maintain their well-being. 

Limitations

The validity of our findings is limited as the nature of the study design was a cross-sectional observational study using an online self-administered questionnaire that was conducted in a single place in Saudi. In addition, the sample size was small. As a result, it is highly recommended that future studies be conducted as generalized studies with larger sample sizes and a more comprehensive investigation of all possible variables that could affect parents’ assessment of QOL. However, the study was strengthened by focusing on a specific, well-defined population of parents with children who have thalassemia using a validated questionnaire. 

## Conclusions

The study showed that parents with thalassemic children exhibited a moderate level of QOL and health in Medina City, Saudi Arabia. The QOL and health of parents of thalassemia children seem to be negatively affected by economic status and expatriation. This indicates that parents of thalassemia children need socioeconomic support in order to maintain a satisfactory QOL and health. Additionally, establishing social support networks and community groups can provide emotional and practical support, while increasing public awareness can reduce stigma and promote empathy for thalassemia.
